# Supplementary material for: Single Cell Transcriptomic and Chromatin Profiles Suggest Layer Vb Is the Only Layer With Shared Excitatory Cell Types in the Medial and Lateral Entorhinal Cortex
Source: Front Neural Circuits. 2022 Jan 26;15:806154. doi: 10.3389/fncir.2021.806154 (PMC8826650; doi:10.3389/fncir.2021.806154)
Supplement: Supplementary file 14 [file Data_Sheet_1.ZIP › LEC_Sup_vs_MEC_Sup_Motifs/homerResults/motif4.similar.html]

motif4

## Information for motif4

A
C
G
T
A
C
T
G
G
A
C
T
A
G
C
T
C
G
T
A
A
C
G
T
A
G
T
C
C
G
T
A
  
Reverse Opposite:  

G
C
A
T
C
T
A
G
C
G
T
A
A
C
G
T
T
C
G
A
C
T
G
A
T
G
A
C
T
G
C
A
  

|  |  |
| --- | --- |
| p-value: | 1e-62 |
| log p-value: | -1.428e+02 |
| Information Content per bp: | 1.668 |
| Number of Target Sequences with motif | 6971.0 |
| Percentage of Target Sequences with motif | 52.28% |
| Number of Background Sequences with motif | 8980.3 |
| Percentage of Background Sequences with motif | 45.07% |
| Average Position of motif in Targets | 322.7 +/- 235.3bp |
| Average Position of motif in Background | 361.4 +/- 288.8bp |
| Strand Bias (log2 ratio + to - strand density) | 0.0 |
| Multiplicity (# of sites on avg that occur together) | 1.41 |
| Motif File: | file (matrix) reverse opposite |

### Similar de novo motifs found

|  |  |  |  |  |  |  |  |
| --- | --- | --- | --- | --- | --- | --- | --- |
| Rank | Match Score | Redundant Motif | P-value | log P-value | % of Targets | % of Background | Motif file |
| 1 | 0.737 | C G T A T A C G C G A T A C T G C G A T C T A G G C T A C G A T G C T A C G T A G T A C G C T A | 1e-25 | -57.911657 | 10.67% | 8.09% | motif file (matrix) |
| 2 | 0.673 | T C G A A G C T G T C A C G T A A T G C C T G A C T A G G A C T | 1e-24 | -56.854796 | 69.91% | 65.70% | motif file (matrix) |
| 3 | 0.782 | T C A G G T C A G A C T C T G A T C G A A T G C T G C A C A G T A G T C C T A G | 1e-18 | -43.707752 | 18.70% | 15.79% | motif file (matrix) |
| 4 | 0.701 | C A G T C T A G T G C A C G A T C G T A G T C A G T A C C T A G C A T G T C G A G T C A C A G T | 1e-12 | -29.016615 | 0.18% | 0.03% | motif file (matrix) |
| 5 | 0.652 | C G T A C A T G C A G T T A C G G C A T T A G C C A G T T A C G C T G A C G A T T C G A T G C A | 1e-8 | -19.565593 | 0.14% | 0.03% | motif file (matrix) |
